# Utilizing a novel fecal sampling method to examine resistance of the honey bee (*Apis mellifera*) gut microbiome to a low dose of tetracycline

**DOI:** 10.1371/journal.pone.0317129

**Published:** 2025-01-16

**Authors:** Casey L. Gregory, Emma L. Bradford, Richard D. Fell, David C. Haak, Lisa K. Belden

**Affiliations:** 1 Department of Biological Sciences, Virginia Tech, Blacksburg, Virginia, United States of America; 2 Department of Entomology, Virginia Tech, Blacksburg, Virginia, United States of America; 3 School of Plant and Environmental Sciences, Virginia Tech, Blacksburg, Virginia, United States of America; Tanta University Faculty of Science, EGYPT

## Abstract

Disruption of host-associated microbial communities can have detrimental impacts on host health. However, the capacity of individual host-associated microbial communities to resist disturbance has not been well defined. Using a novel fecal sampling method for honey bees (*Apis mellifera*), we examined the resistance of the honey bee gut microbiome to disruption from a low dose of the antibiotic, tetracycline (4.5 μg). Prior to the experiment, bacterial communities from fecal samples were compared to communities from dissected whole guts of the same individuals to ensure fecal samples accurately represented the gut microbiome. Fecal samples were collected from lab-caged honey bees prior to, and five days after, tetracycline exposure to assess how antibiotic disturbance affected the communities of individuals. We used metrics of alpha and beta diversity calculated from 16S rRNA gene amplicon sequences to compare gut community structure. Low dose tetracycline exposure did not consistently change honey bee gut microbiome structure, but there was individual variation in response to exposure and specific taxa (one ASV assigned to *Lactobacillus kunkeei* and one ASV in the genus *Bombella*) were differentially abundant following tetracycline treatment. To assess whether individual variation could be influenced by the presence of tetracycline resistance genes, we quantified the abundance of tet(B) and tet(M) with qPCR. The abundance of tet(M) prior to tetracycline treatment was negatively correlated with change in community membership, assessed by difference in Jaccard dissimilarity over the five-day experiment. Our results suggest that the honey bee gut microbiome has some ability to resist or recover from antibiotic-induced change, specific taxa may vary in their susceptibility to tetracycline exposure, and antibiotic resistance genes may contribute to gut microbiome resistance.

## Introduction

Almost all plants and animals have host-specific microbial communities that reside on and within their bodies. These communities of microorganisms are often termed “microbiomes’’, and consist of bacteria, fungi, and viruses. In many organisms, the microbiome is essential to the health and development of the host. The microbiome of the gastrointestinal tract is of particular importance due to its extensive impacts on host health [1–3], including facilitating the breakdown of indigestible materials, aiding in the absorption of nutrients, regulating the host immune response, and stimulating growth and development [2–5]. In addition, the gut microbiome can benefit the host by deterring pathogen invasion. Members of the microbiome can prevent the establishment of pathogens and parasites by producing toxins that inhibit pathogen growth, outcompeting pathogens for resources, stimulating the host’s innate immune response, and altering the host environment to create unfavorable conditions for pathogens [6–8]. Disruption of the natural gut microbiota can have negative effects on hosts by interfering with these functions. For example, organisms with absent or disrupted gut bacterial communities tend to have higher pathogen loads [9,10].

An array of factors have the potential to alter normal gut communities, including pesticides, pathogens, and antibiotic exposure [11–13]. Antibiotics are widely used in medical and agricultural settings [14,15], and are also environmental contaminants [16]. Broad spectrum antibiotics have been vital for improving outcomes for diseases caused by bacterial pathogens. However, the mis- or overuse of antibiotics may cause irreversible damage to host-associated microbial communities, and ultimately have negative effects on host health. For example, studies on both mice and insects suggest antibiotics may impair neurological function by disrupting the gut-brain axis [17,18].

Understanding to what extent host-associated bacterial communities can resist or recover from antibiotic disturbance could help inform decisions on when, and in what quantities, antibiotics can safely be used. Drawing from ecological principles of disturbance and recovery can help define community response to disturbance. Ecological disturbance has been defined as a discrete event “that disrupts the structure of an ecosystem, community, or population, and changes resource availability or the physical environment” [19]. When confronted by a disturbance event, communities may resist change (resistance), change temporarily and then return to the pre-disturbed state (resilience), or change permanently [20]. A community’s capacity for resistance and resilience is known as community stability [20,21]. In the case of antibiotic disturbance of a bacterial community, we can define the discrete event as the duration of antibiotic exposure, and the disruption in structure as a change in the abundance or diversity of the bacterial taxa that make up the community. In this study, we used a single low dose of tetracycline, a common broad-spectrum antibiotic, as our disturbance event, and examined whether this event brought about change in the honey bee gut microbiome.

*Apis mellifera*, the European honey bee, is economically important, contributing $6.4 billion annually to the USA economy through pollination services [22]. Increases in yearly losses of managed honey bee colonies have raised concerns about food security. The cause of elevated colony losses appears to be the synergistic interaction of several factors, including climate change, poor nutrition, land management practices and disease [23,24]. A contributor to reduced honey bee health is disruption of the symbiotic bacterial communities that reside within the guts of adult bees (the gut microbiome) [25]. Therefore, research on disturbance and resistance of the honey bee gut microbiome may provide insight into how to improve the health of managed honey bees.

The adult honey bee gut microbiome is made up of eight to ten bacterial phylotypes [25–27] that perform essential functions, such as breaking down otherwise indigestible compounds [28], stimulating individual immune response [29], and preventing pathogen establishment [9]. Bees with disrupted or absent gut microbes experience higher mortality [30] and have higher pathogen loads [9]. As in other animals, antibiotics can lead to disruption of the honey bee gut microbiome [9,30–32]. Oxytetracycline is used by some beekeepers to treat bacterial brood diseases caused by *Paenibacillus larvae* and *Melissococcus plutonius* [18,33,34]. A typical oxytetracycline treatment in hives consists of three 200 mg applications 2–5 days apart [35,36]. While tetracycline in hives is meant to treat infected brood, adult bees may ingest the antibiotic by cleaning hive surfaces, grooming nestmates, or consuming contaminated honey [37].

Understanding how sensitive the honey bee gut microbiome is to tetracycline is important, as disturbance of the honey bee gut microbiome with tetracycline negatively impacts honey bee fitness. In adult honey bees, tetracycline can increase lipid concentration and alter normal development [18]. Tetracycline can also alter learning and memory, as certain members of the microbiome (i.e. *Lactobacillus*) are required for regulating metabolism of compounds essential to neurological processes [38]. Additionally, tetracycline alteration of the honey bee gut microbiome can exacerbate honey bee diseases [30,39], which are major promoters of colony loss [23,24]. These studies highlight the potential negative effects of prolonged tetracycline use in hives, and the importance of examining the response of bee associated bacterial symbionts to tetracycline exposure.

Laboratory studies assessing the effects of tetracycline of the honey bee gut microbiome typically provide bees with *ad libitum* tetracycline solution at 450 μg/mL for 5 days [30–32,38–41]. At this dose, tetracycline reduces the abundance of core honey bee gut symbionts and alters gut community structure [30,31,38–41]. However, one laboratory study that used a lower dose of tetracycline (10 μg/mL) and a semi-field study that treated micro-hives with 13.5 mg of tetracycline, found minimal effects of tetracycline on gut community structure [31,32]. This could be caused by bees ingesting different quantities of tetracycline or by variation in the resistance of individual gut communities. In our study, we individually fed bees 4.5 μg of tetracycline to ensure they all received the same dose.

We chose a relatively low dose of tetracycline (10 μL of the standard 450 μg/mL) to determine if a single, low dose ingestion of tetracycline could alter the honey bee gut microbiome of individual bees. To confirm that fecal samples accurately portray the whole gut microbiome, we first compared the bacterial communities from fecal and whole gut samples using 16S rRNA amplicon sequencing. After verifying our methodology, we carried out an experiment in which fecal samples were collected from individual adult worker honey bees prior to and five days after treatment with 4.5 μg of tetracycline. In addition to analyzing compositional changes in the gut bacterial community, we explored how genetic components of the honey bee gut microbiome influence community stability and respond to disturbance events. In particular, we compared the abundance of two tetracycline resistance genes, tet(B) and tet(M), before and after tetracycline treatment. We chose these genes because they have been identified previously in the honey bee gut microbiome [34]. By analyzing the abundance of tet(B) and tet(M), we sought to identify whether gut communities with higher abundances of resistance genes were better able to resist tetracycline disturbance.

The overall aim of this study was to 1.) demonstrate the validity of a new fecal sampling method for honey bees, 2.) analyze the compositional stability of the honey bee gut microbiome to a low dose of tetracycline, and 3.) explore how antibiotic resistance genes influence community stability. In the presented work, we successfully show that fecal samples from honey bees convey the composition of individual honey bee gut microbiota. In addition, we demonstrate that the honey bee gut microbiome may be compositionally stable in response to low doses of tetracycline. However, gut communities from individual bees, as well as certain bacterial taxa, may vary in their resistance to tetracycline. Finally, our results suggest tet(M) abundance prior to tetracycline exposure may contribute to community response to tetracycline, and certain taxa may have a higher propensity for harboring antibiotic resistance genes.

## Materials and methods

Honey bees were collected from hives managed by Virginia Tech faculty and located on Virginia Tech property. No permits were required to access field sites or collect samples.

### Assessment of fecal sample vs. whole gut microbiome

#### Honey bee collection and study design

To test the efficacy of fecal sampling, seven adult honey bee workers were collected from a single hive located at the Virginia Tech Turf Grass Research Center in Blacksburg, VA USA, and returned to the laboratory. All seven workers were taken from the center of the hive body, and placed into a sterile collection container for transport to the laboratory. Immediately upon returning from the hives, fecal samples were collected from each live bee by gently pinching the abdomen until defecation occurred into a sterile microcentrifuge tube. Clean gloves and sterile forceps were used to handle each bee. Each sample was immediately treated with lysozyme (20 mg lysozyme per 1 mL lysis buffer) in lysis buffer (180 μL, containing 20 mM Tris-HCL pH 8, 2 mM EDTA pH 8, and 1.2% Triton-x-100). The bees from which the fecal samples were collected were then euthanized at -20°C. Once the bees were euthanized, their entire intestinal tracts (from honey stomach to rectum) were dissected and individually placed into sterile microcentrifuge tubes containing lysis buffer with lysozyme (as above).

#### DNA extraction

DNA was extracted from the paired fecal and whole gut samples (N = 14 samples; 7 bees). Fecal samples and whole guts were ground for 5 seconds in the lysozyme pre-treatment using sterile plastic pestles, and then incubated at 37°C for 1 hour. DNA was extracted from the samples using the Qiagen DNeasy Kit (Qiagen, Germantown, MD, USA) following manufacturer’s instructions, with a final elution in 50 μL molecular grade water.

#### 16S rRNA gene amplicon sequencing

PCR was performed to amplify the V4 region of the 16S rRNA gene using 515F and individually-barcoded 806R primer pairs developed by Caporaso et al. [42]. Each 25 μL reaction included: 0.5 μL of the forward and reverse primers, 9.5 μL UltraClean PCR grade H_2_O, 12.5 μL Promega GoTaq G2 colorless Master Mix, and 2 μL sample DNA. Thermal cycler conditions were: 94°C for 3 min, followed by 34 cycles of 94°C for 45 sec, 50°C for 60 s, 72°C for 90 s, and a final extension at 72°C for 10 min. PCR products were quantified using a Qubit 2.0 fluorometer, and 200 ng of DNA from each sample was pooled. The 14 samples from this fecal-sampling study were included on the same sequencing run as the samples from the tetracycline experiment. The pooled sample was cleaned using the QIAquick PCR purification kit (Qiagen, Inc., Valencia, CA) following manufacturer’s instructions, and eluted in 50 μL buffer EB (10 mM Tris Cl, pH 8.5). Barcoded amplicons were sent to the Molecular Biology Core Facilities of the Dana Farber Cancer Institute at Harvard University (Cambridge, MA USA) for sequencing on an Illumina MiSeq instrument using a 250 base pair single-end strategy.

Raw single-end 16S rRNA amplicon sequences were processed with the Qiime2 pipeline v.2019.1 [43]. In DADA2 [44], the sequences were demultiplexed and denoised. For denoising, our quality score parameter was set to 11, and reads were truncated to 250 bp. After demultiplexing, we had a total of 4737603 read with a range of 49744–169727 reads per sample. After denoising, we had 306 ASVs across 40 samples with a range of 34392–117727 reads/sample. Reads with a frequency of less than 0.01% were then filtered out across all samples. After filtering, we had 58 ASVs across 40 samples with a range of 31351–117180 reads/sample. These 58 ASVs were assigned taxonomy using the SILVA database (v.13.2) [45] trained for use with DADA2. RAxML v.8 [46] was used to construct our tree, which was rooted at the midpoint. We set our p seed to 1723, rapid bootstrap seed to 9384, replicates to 100, and substitution model to GTRCAT. The resulting ASV table, taxonomy table, and phylogenetic tree were imported into R (v.4.3.1) [47]. Before analysis, ASVs classified as chloroplast or mitochondria sequences were filtered from the ASV table. ASVs were further filtered to include only sequences identifiable at the phylum level and belonging to the kingdom Bacteria. Our final dataset for the fecal vs. whole gut comparison contained 39 unique ASVs across 14 samples.

#### Statistical analysis

Statistical analyses were conducted in R v.4.3.1 [47]. To determine whether the bacterial communities from our fecal samples accurately represented the whole honey bee gut microbiome, we compared the communities using Bray Curtis [48] and Jaccard [49] dissimilarity. Bray Curtis dissimilarity quantifies community differences based on the relative abundances of the taxa, while Jaccard dissimilarity accounts for the presence or absence of taxa. To calculate Bray Curtis and Jaccard dissimilarity, we first normalized our data by converting the raw ASV counts to proportions. We then generated a Bray Curtis dissimilarity matrix and a Jaccard dissimilarity matrix using the ‘ordinate’ function in Phyloseq (v.1.38.0) [50], and visualized them using NMDS ordinations. A PERMANOVA (function adonis2; vegan package v2.5–7, [51]) based on 999 permutations was used to analyze group-level differences in Bray Curtis and Jaccard dissimilarity of the bacterial communities from the whole gut and fecal samples. We then extracted the Bray Curtis and Jaccard dissimilarity values from paired fecal and gut samples (collected from the same bee) and compared them to unpaired gut and fecal samples (collected from different bees) using a two-sample t-test.

To determine whether certain taxa were over- or underestimated in the fecal samples compared to the whole gut samples, we performed differential abundance analysis with ALDEx2 on the ASVs and the ASVs sorted into genera [52,53]. We used the aldex.clr function to generate Monte Carlo samples of the Dirichlet distribution for each sample and perform a centered log- ratio transformation on each instance. Welch’s t-tests were calculated from the transformed values using aldex.ttest with the paired.test argument set to true. The Benjamini-Hochberg procedure was used for *p*-value adjustment to control for false discoveries. Taxa were considered differentially abundant if, after adjustment, *p*-values were <0.05. Effect sizes were calculated with aldex.effect, and an effect size cutoff of >1 was used. Column descriptions for ALDEx2 outputs are provided in (S1 Table).

### Assessment of tetracycline disturbance using fecal samples

#### Honeybee collection and experimental design

Two frames of capped brood were collected from a single hive located at the Virginia Tech Turf Grass Research Center, Blacksburg, VA USA in April 2020. The frames were maintained at 32°C overnight inside emergence cages to allow bees to emerge from the capped cells. All bees used in this experiment emerged less than 24 hours after the frames were collected from the hive. Approximately 300 of the newly emerged bees were briefly sedated with CO_2_ and marked on the thorax with a blue dot (bullet tip medium line Uni Pasca paint pen). Marked bees were returned to their hive of origin for 7 days to allow the gut microbiome to establish [54]. After 7 days, marked bees were recollected for the experiment using sterile forceps.

Upon return to the laboratory, bees were divided into 6 plastic 9x9x8cm hoarding cages with 20–30 bees/cage [55] and each cage was randomly assigned to an antibiotic or control treatment (N = 3 cages/treatment). Focal bees from each cage were randomly selected to receive treatment. Prior to treatment, fecal samples were collected from all focal bees into 1.5 mL microcentrifuge tubes and stored at -70°C until processing. Focal bees were then individually-marked with color-coded numbered discs, so individuals could be identified. After fecal sampling and marking, focal bees were placed in individual feeding chambers (made from 0.5 mL microcentrifuge tubes with the bottoms cut off) and fed 10 μl of treatment solution using a micropipette. Bees that did not consume the entire treatment bolus were not included in the study. Control focal bees were fed 10 μl of a 50% w:v sucrose solution, and antibiotic focal bees were fed 10 μl of a 450 μg/ml tetracycline solution dissolved in 50% w:v sucrose. All treatment solutions were prepared using sterile water. Focal bees were isolated from cage mates in their feeding chambers for 30 min after treatment to reduce the likelihood of the focal bees transferring the treatment via trophallaxis (exchange of food by mouth) [56]. After 30 min, the focal bees were returned to the cages with untreated nestmates. Bees were maintained in the hoarding cages for 5 days and were provided 50% w:v sucrose in sterile water *ad libitum* from feeding tubes modified from microcentrifuge tubes. Cages were checked daily, and dead bees were removed. Focal bees that died before the 5 day experiment was complete (N = 23) were not included in the final analysis. Fecal samples were collected again from focal bees five days after treatment (N = 7 controls, 6 antibiotic-treated) and stored at -70°C until further processing.

#### DNA extraction and 16S rRNA gene amplicon sequencing

DNA extractions and amplicon PCR were completed from the fecal samples as described for the preliminary comparison of fecal vs. whole gut samples. A total of 26 samples from this experiment (antibiotic day 0 N = 6, antibiotic day 5 = 6, control day 0 = 7, control day 5 = 7) were sequenced as part of the same run as our whole gut and fecal sample comparison, and prepped as described previously. Additionally, we used the same methods to process the bacterial community data in QIIME2, and the processing summary is presented above in the fecal/whole gut methods [43]. Our final dataset for the antibiotic trial contained 49 unique ASVs across 26 samples.

#### qPCR of tetracycline resistance genes tet(B) and tet(M)

We quantified the tetracycline resistance genes tet(B) and tet(M), as they have been previously detected in honey bee gut bacteria [34]. For these assays, we used primers created by Aminov et al. [57–59] and developed gBlock® gene fragment (Integrated DNA Technologies, Coralville, IA USA) standards (Table 1). Standard curves were made for each run of the SYBR assays using 10-fold dilutions (10^9^ to 10^3^ gene copies) of the gBlock® gene fragments (Integrated DNA Technologies, Coralville, IA USA). Each reaction was 15 μl, and contained 3 μl molecular water, 7.5 μl SSoAdvanced™ Universal SYBR® Green Supermix (Bio-Rad Labs, Hercules, CA USA), 0.75 μl of each primer (10μM), and 3 μl template DNA or gBlock® standard. Samples, standards, and no template controls were run in triplicate and the average of the triplicates was used in analyses. Assays were run on a CFX96 Touch™ Real-Time PCR Detection System (Bio-Rad Labs, Hercules, CA USA). The tet(B) reaction used the conditions: 94°C for 1 min, followed by 45 cycles of 94°C for 30 sec, 65°C for 1 min, and 72°C for 1 min [58,59]. The tet(M) reaction used the conditions: 94°C for 10 min, followed by 45 cycles of 94°C for 15 sec, 55°C for 1 min, and 61°C for 1 min [57]. The number of gene copies in each sample was calculated based on the run’s standard curve. Tet(B) and tet(M) copy number were standardized by dividing gene copy number by the total amount of DNA in each 3 μl sample.

**Table 1:** Tetracycline resistance gene qPCR primer sequences.

	Forward Primer	Reverse Primer	Reference	gBlock® standard
Tet(B)	TACGTGAATTTATTGCTTCGG	ATACAGCATCCAAAGCGCAC	Aminov (2002), [59] Aminov (2004) [58]	CCCAGTACGTGAATTTATTGCTTCGGTAGGGAATCTTCCGCAATGGACGAAAGTCTGACGGAGCAACGCCGCGTGAGTGATGAAGGTTTTCGGATCGTAAAGCTCTGTTGTTAGGGAAGAACAAGTGCAAGAGTAACTGCTTGCACCTTGACGGTACCTAACCAGAAAGCCACGGCTAACTACGTG ATACAGCATCCAAAGCGCACACGTAGG
Tet(M)	ACAGAAAGCTTATTATATAAC	TGGCGTGTCTATGATGTTCAC	Aminov (2001) [57]	CCCAGACAGAAAGCTTATTATATAACTAGGGAATCTTCCGCAATGGACGAAAGTCTGACGGAGCAACGCCGCGTGAGTGATGAAGGTTTTCGGATCGTAAAGCTCTGTTGTTAGGGAAGAACAAGTGCAAGAGTAACTGCTTGCACCTTGACGGTACCTAACCAGAAAGCCACGGCTAACTACGTGTGGCGTGTCTATGATGTTCACACGTAGG

Sequences of the primers and gBlock® standards used for the tet(B) and tet(M) qPCR assays.

#### Statistical analysis

The ASV table, taxonomy table, and phylogenetic tree for the tetracycline experiment were imported into R (v.4.1.2) [47] for analysis. Raw ASV counts were converted to proportions (relative abundance) and used to assess community diversity.

We first hypothesized that antibiotic exposure would cause quantifiable changes to the gut communities compared to the control bees. To test this, Bray Curtis and Jaccard dissimilarity were calculated for each fecal community. Separate PERMANOVAs were performed for the antibiotic and control groups. We also extracted the dissimilarity values comparing the dissimilarity of the gut communities of individual bees on day 0 and day 5. These values were then separated by treatment, and the mean values were compared using a Two Sample t-test.

We also hypothesized that the honey bees may possess gut bacterial communities with differential ability to resist antibiotic induced change. Therefore, we tested for differences in group dispersion between day 0 and day 5 in the control and antibiotic treated bees using the betadisper() function in vegan v2.5–7 [51]. If our hypothesis was correct, we expected to observe significant differences in dispersion from day 0 to day 5 in the antibiotic-treated bees, but not in the control bees. An analysis of variance (ANOVA) was performed to test for statistically significant differences in dispersion. ANOVAs were performed based on treatment group.

In addition, we hypothesized that the antibiotic treated bees would experience a decrease in gut bacterial diversity from day 0 to day 5. To test this hypothesis, we calculated three alpha diversity metrics that account for different aspects of community composition for each fecal sample and compared them on day 0 and day 5. The alpha diversity metrics used were richness, phylogenetic diversity, and Inverse Simpson. Richness measures the number of ASVs per sample (corresponds to number of taxa in each community), Faith’s phylogenetic diversity accounts for the relatedness of taxa, and Inverse Simpson quantifies diversity based on richness and evenness. We expected the antibiotic treated bees to experience a greater reduction in richness, phylogenetic diversity and evenness (since the antibiotics would disrupt the ratios of core phylotypes, allowing taxa with greater antibiotic resistance to dominate the community) from day 0 to day 5 than the control bees. To test this, we subtracted the day 5 fecal sample alpha diversity metrics from the day 0 fecal sample alpha diversity metric. We then compared the mean change in diversity from day 0 to day 5 in the antibiotic and control groups using a two-sample t test. Negative values indicate a decrease in diversity and evenness from day 0 to day 5, while positive values would indicate an increase in diversity and evenness from day 0 to day 5. Therefore, we expected the antibiotic-treated bees to have a lower mean change than the control bees. We also compared the mean alpha diversity metrics from day 0 to day 5 in the antibiotic and control bees using paired two-sample t-tests. Before all t-tests were performed, we determined whether the variances of the groups were equal using f-tests. If the variances were equal, the pooled variance was used to estimate the variance. If the variances were not equal, the Welch approximation to the degrees of freedom was used.

We anticipated taxa within the gut would be differentially impacted by tetracycline treatment. Therefore, we performed differential abundance analyses on the complete set of unique ASVs, and on ASVs clustered at the genus level, using ALDEx2, as described for our fecal/whole gut comparison. These analyses were performed on the antibiotic treated bees, and compared across collection day.

We predicted tetracycline exposure would select for tetracycline resistance genes in the communities, and that variation in gut microbiome resistance may be influenced by the presence of tetracycline resistance genes. To examine whether tetracycline exposure resulted in accumulation of tetracycline resistance genes in the communities, we compared the abundance of tet(B) and tet(M) in fecal samples before and after tetracycline exposure. One-way ANOVAs were used to compare mean resistance gene abundance on experimental day 0 (before tetracycline exposure) and day 5 (after tetracycline exposure). Separate tests were performed for each of the two genes (tet(B) and tet(M)) and treatments (antibiotic and control). We anticipated that tet(B) and tet(M) would be significantly more abundant after tetracycline treatment (day 5). To test whether variation in community stability was caused by tetracycline resistance genes, we examined whether resistance gene abundance prior to tetracycline exposure predicted community change. We measured community change using Bray Curtis and Jaccard distance values comparing individuals on day 0 and day 5. If the distance is zero, it indicates identical communities on day 0 and day 5; as the value increases it means the communities are more dissimilar. We then used linear models (estimated using OLS) to predict the magnitude of community change based on resistance gene abundance. Individual models were run for each gene (tet(B) and tet(M)) and treatment group (antibiotic and control). We expected tet(B) and tet(M) abundance on day 0 to be negatively correlated with Bray Curtis and Jaccard distance, indicating that higher abundance of tetracycline resistance genes at day 0 would result in less change in community structure following tetracycline exposure.

## Results

### Fecal sample vs. whole gut microbiome comparison

**Fig 1:**
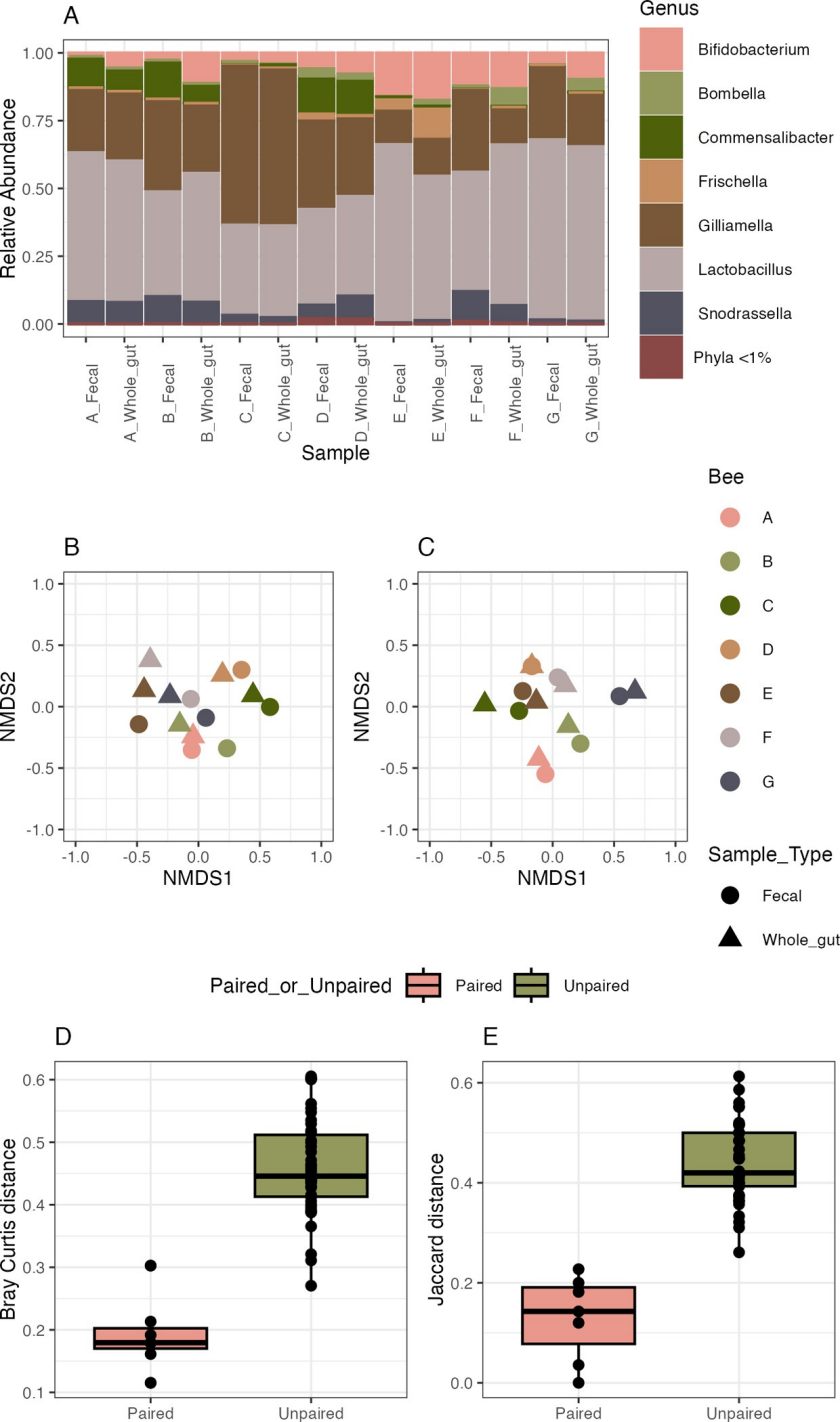
Comparisons of the bacterial communities from whole gut and fecal samples. Samples were collected in April 2020. A) Relative abundance of the bacterial genera in the fecal samples and whole dissected gut of individual adult worker honey bees separated by sample type. NMDS ordinations of B) Bray Curtis and C) Jaccard dissimilarity calculated from the bacterial communities of fecal and whole gut samples of seven worker honey bees. Median with 1st and 3rd quartiles of Bray Curtis (D) and Jaccard (E) dissimilarity values of paired individual fecal and whole samples and unpaired samples. Dots are individual data points.

While there was individual variation in relative abundances, the fecal and whole gut communities were dominated by genera associated with the honey bee gut microbiome including *Bifidobacterium*, *Frischella*, *Gilliamella*, *Lactobacillus*, and *Snodgrassella* (Fig 1A). ASVs corresponding to the core members of the honey bee gut microbiome made up 95% of the communities. Bacterial communities from feces and whole guts did not differ based on sample type when assessed by Bray Curtis or Jaccard dissimilarity (Fig 1B and 1C; Bray-Curtis, PERMANOVA, df = 1, ss = 0.05149, R^2^ = 0.03986, F = 0.4982, *p* = 0.882 | Jaccard, PERMANOVA, df = 1, ss = 0.016, R^2^ = 0.01321, F = 0.1607, *p* = 0.973). We also examined the average difference in Bray Curtis and Jaccard dissimilarities between gut and fecal samples from the same bee (paired) and gut and fecal samples taken from different individuals (unpaired) (Fig 1D and 1E). Both Bray Curtis and Jaccard dissimilarity were significantly lower in the paired samples compared to the unpaired samples, indicating that the bacterial communities in the fecal and gut samples taken from the same bee were more alike in community structure and composition than those from different bees (Fig 1D and 1E; Bray Curtis, two sample t-test, difference = -0.27, 95% CI [-0.33, -0.21], t(47) = -8.64, *p* < .001; Cohen’s d = -2.52, 95% CI [-3.28, -1.75] | Jaccard, two sample t-test, difference = -0.31, 95% CI [-0.38, -0.25], t(47) = -9.57, *p* < .001; Cohen’s d = -2.79, 95% CI [-3.59, -1.98]).

None of the 39 unique ASVs or 10 genera detected in the fecal and whole gut samples were differentially abundant based on sample type.

### Assessment of tetracycline disturbance using fecal samples

**Fig 2:**
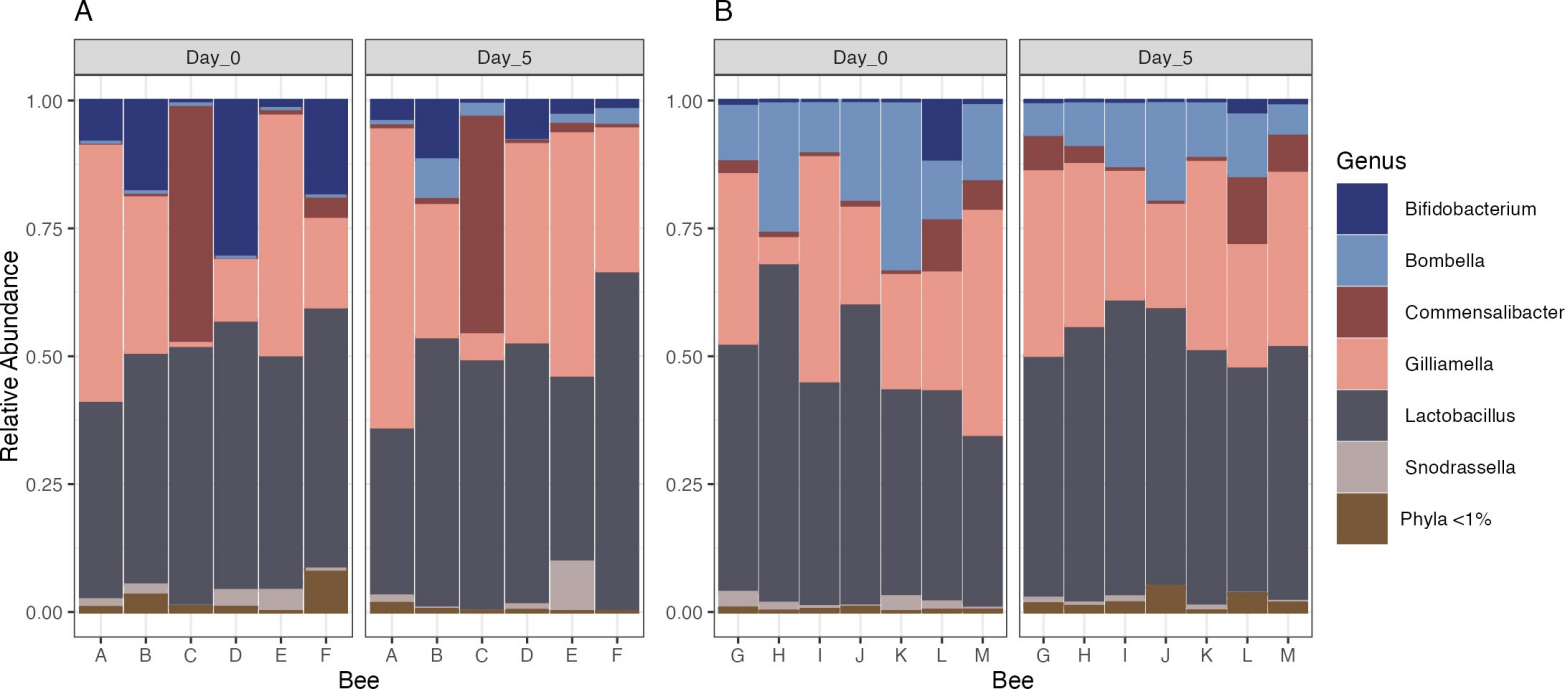
Relative abundance of the bacterial genera in the fecal samples of A) antibiotic treated and B) control bees before (Day 0) and five days after treatment (Day 5).

**Fig 3:**
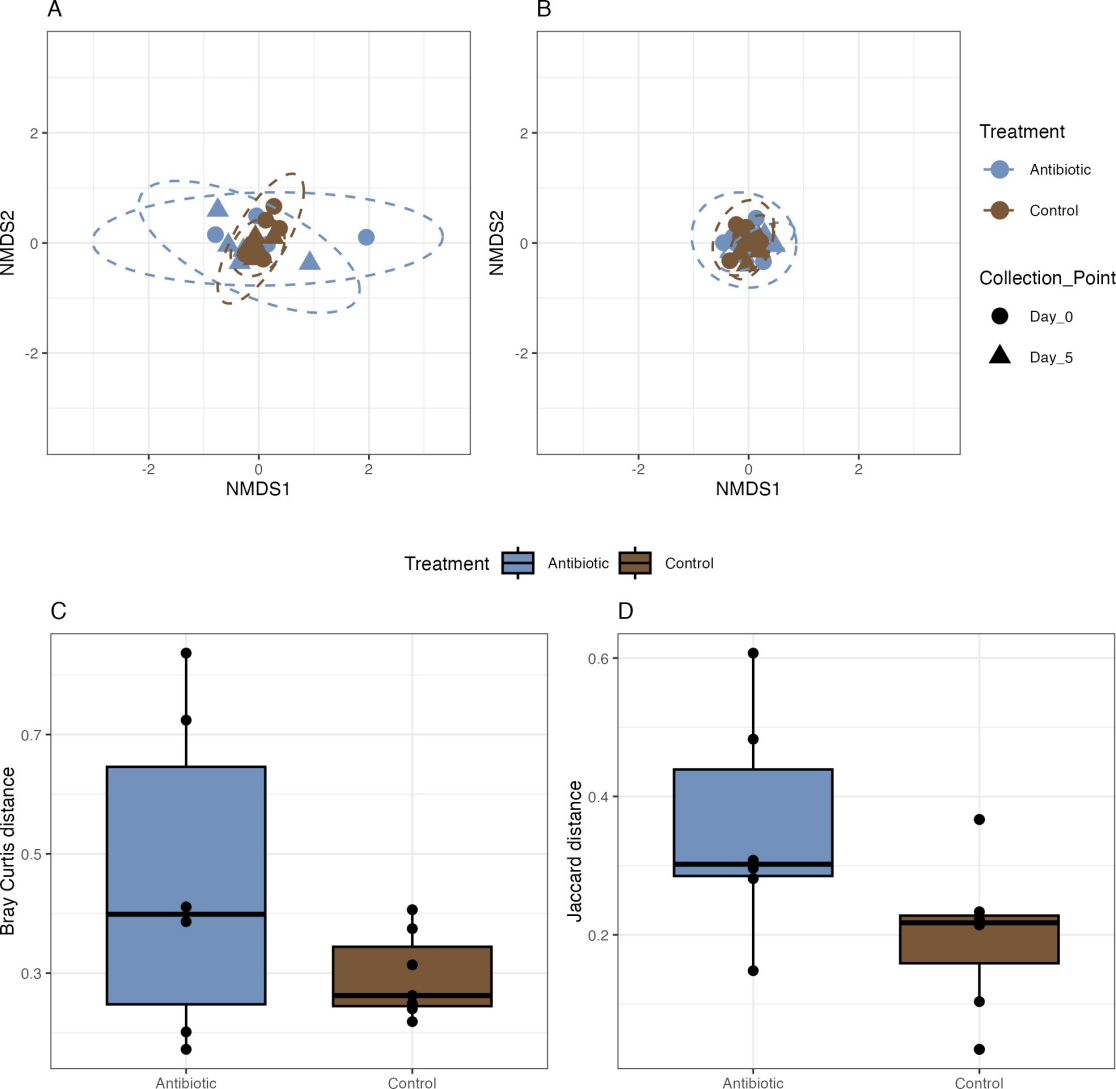
Comparison of honey bee gut bacterial communities treated with sucrose containing antibiotics (Antibiotic) or plain sucrose (Control) before (Day 0) and five days after treatment (Day 5). A) Bray Curtis NMDS ordination B) Jaccard NMDS ordination C) Median and 1st and 3rd quartiles of change in Bray Curtis dissimilarity from day 0 to day 5 in the antibiotic and control fecal communities. D) Median and 1st and 3rd quartiles of change in Jaccard dissimilarity from day 0 to day 5 in the antibiotic and control fecal communities.

The majority of the ASVs (96%) in both the control and antibiotic-treated bees corresponded to bacterial taxa typical of the honey bee gut microbiome, including the genera *Bifidobacterium*, *Gilliamella*, *Lactobacillus*, and *Snodgrassella* (Fig 2). The control communities did not differ significantly between day 0 and day 5 based on Bray Curtis or Jaccard dissimilarity (Fig 3A and 3B; Bray-Curtis, PERMANOVA, df = 1, ss = 0.22381, R^2^ = 0.09909, F = 1.0999, *p* = 0.408 | Jaccard, PERMANOVA, df = 1, ss = 0.08360, R^2^ = 0.10383, F = 1.3903, *p* = 0.266). The fecal communities of the antibiotic-treated bees also did not differ between day 0 and day 5 based on Bray Curtis dissimilarity, but did differ based on Jaccard dissimilarity (Fig 3A and 3B; Bray-Curtis, PERMANOVA, df = 1, ss = 0.10919, R^2^ = 0.09819, F = 1.3065, *p* = 0.219 | Jaccard, PERMANOVA, df = 1, ss = 0.16158, R^2^ = 0.15928, F = 1.8945, *p* = 0.004). Mean Bray Curtis dissimilarity of individuals from day 0 to day 5 was not significantly different between the control and antibiotic treatment groups (Fig 3C; Bray-Curtis, two sample t-test, difference = 0.16, 95% CI [-0.12, 0.44], t(5.60) = 1.40, *p* = 0.213; Cohen’s d = 1.19, 95% CI [-0.65, 2.93]). Likewise, mean Jaccard dissimilarity of individuals on day 0 and day 5 did not differ significantly between the control and antibiotic treatment groups (Fig 3D; Jaccard, two sample t-test, difference = 0.16, 95% CI [-0.01, 0.32], t(11) = 2.06, *p* = 0.063; Cohen’s d = 1.25, 95% CI [-0.07, 2.51]). We measured treatment group dispersion to test whether individual honey bee gut communities would express differential ability to resist or recover from antibiotic induced changes in bacterial relative abundance and presence absence. Control group dispersion did not significantly differ based on Bray Curtis and Jaccard dissimilarity between days 0 and 5 (Bray Curtis, ANOVA, df = 1, ss = 0.022323, R^2^ = 0.0223228, F = 4.6011, *p* = 0.05311 | Jaccard, ANOVA, df = 1, ss = 0.000831, R^2^ = 0.0008313, F = 0.1202, *p* = 0.7348). Antibiotic-treated group dispersion also did not significantly differ based on Bray Curtis and Jaccard dissimilarity between days 0 and 5 (Bray-Curtis, ANOVA, df = 1, ss = 0.01482, R^2^ = 0.014821, F = 0.3974, *p* = 0.5426 | ANOVA, df = 1, ss = 0.003645, R^2^ = 0.0036445, F = 0.6285, *p* = 0.4463).

**Fig 4:**
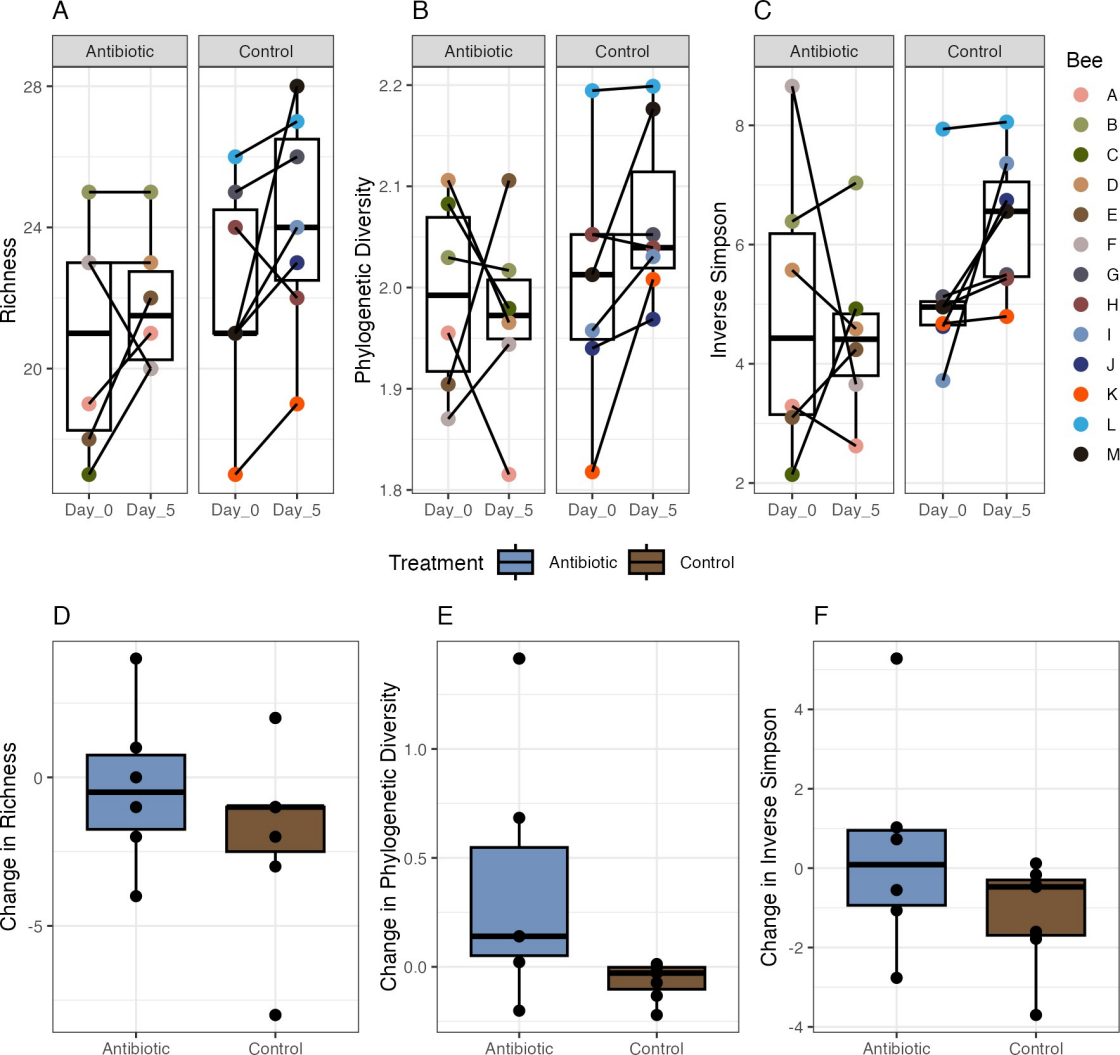
Comparisons of alpha diversity metrics in the fecal communities of honey bees treated with antibiotics and controls before and after treatment. Median, 1^st^, and 3^rd^ quartiles are shown in each boxplot. A-C) Mean A) richness, B) phylogenetic diversity, and C) Inverse Simpson on day 0 and day 5 in the antibiotic and control bees. Values for fecal communities from the same individual are designated by color and connected by lines. D-E) Mean change in fecal community for individual bees (day0—day5 value) in the antibiotic and control treatments assessed as D) richness, E) phylogenetic diversity, and F) Inverse Simpson.

In the fecal communities from the control bees, richness, phylogenetic diversity, and Inverse Simpson did not change significantly from day 0 to day 5 (Fig 4A and 4C; richness, paired t-test, difference = -2.00, 95% CI [-4.83, 0.83], t(6) = -1.73, *p* = 0.134; Cohen’s d = -0.71, 95% CI [-1.57, 0.21] | phylogenetic diversity, paired t-test, difference = -0.06, 95% CI [-0.14, 0.02], t(6) = -1.96, *p* = 0.097; Cohen’s d = -0.80, 95% CI [-1.69, 0.14] | Inverse Simpson, paired t-test, difference = -1.15, 95% CI [-2.38, 0.09], t(6) = -2.28, *p* = 0.063; Cohen’s d = -0.93, 95% CI [-1.85, 0.05]). The fecal communities of the antibiotic-treated bees also did not change significantly in richness, phylogenetic diversity, or Inverse Simpson from day 0 to day 5 (Fig 4A and 4C; richness, paired t-test, difference = -0.33, 95% CI [-3.20, 2.53], t(5) = -0.30, *p* = 0.777; Cohen’s d = -0.13, 95% CI [-1.01, 0.75] | phylogenetic diversity, paired t-test, difference = 0.37, 95% CI [-0.25, 0.99], t(5) = 1.52, *p* = 0.189; Cohen’s d = 0.68, 95% CI [-0.31, 1.62] | Inverse Simpson, paired t-test, difference = 0.44, 95% CI [-2.43, 3.31], t(5) = 0.40, *p* = 0.708; Cohen’s d = 0.18, 95% CI [-0.71, 1.05]). We also examined if there was a difference in the mean change in diversity from day 0 to day 5 between the control and antibiotic groups. We found that mean change did not differ significantly between treatments for richness, phylogenetic diversity, or Inverse Simpson (Fig 4D and 4F; richness, two sample t-test, difference = 1.67, 95% CI[-1.90, 5.23], t(11) = 1.03, *p* = 0.326; Cohen’s d = 0.62, 95% CI [-0.60, 1.82] | phylogenetic diversity, two sample t-test, difference = 0.43, 95% CI [-0.19,1.05], t(5.18) = 1.77, *p* = 0.135; Cohen’s d = 1.55, 95% CI [-0.45, 3.45] | Inverse Simpson, two sample t-test, difference = 1.59, 95% CI [-0.97, 4.15], t(11) = 1.37, *p* = 0.199; Cohen’s d = 0.82, 95% CI [-0.42, 2.04]).

According to our differential abundance analyses, one ASV and one genus were differentially abundant between day 0 and day 5 in the tetracycline treated bees (S2 and S3 Tables). The relative abundance of ASV11, which was classified as *Lactobacillus kunkeei*, was more abundant on experimental day 5 (after tetracycline treatment) than day 0 (ALDEx2, median clr day 0 = -4.75, median clr day 5 = 5.64, diff. btw = 10.6, *p* < 0.001, adj *p* = 0.03, effect size = 1.96). The relative abundance of the genus *Bombella* was also higher on day 5 compared to day 0 in the antibiotic treated bees (ALDEx2, median clr day 0 = -6.73, median clr day 5 = 4.89, diff. btw = 10.5, *p* = 0.001, adj *p* = 0.02, effect size = 2.23).

**Fig 5:**
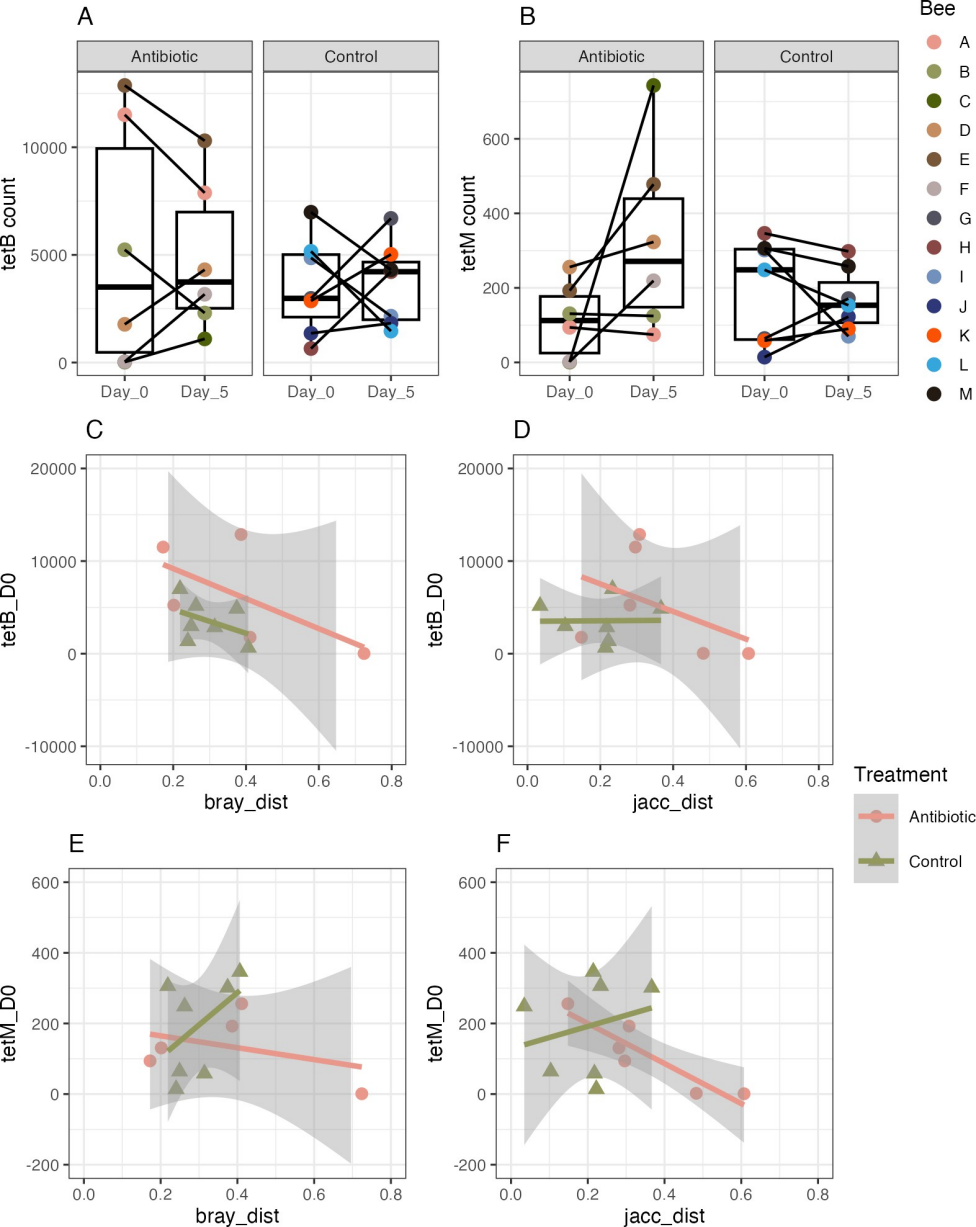
Concentrations of the tetracycline resistance genes tet(B) and tet(M) determined with qPCR and their relation to change in gut community composition. A) Abundance of tet(B) in the gut communities of honey bees before treatment (Day 0) and five days after tetracycline treatment (Day 5) compared with controls. B) Abundance of tet(M) before and five days after tetracycline treatment compared with controls. C) Abundance of tet(B) prior to tetracycline treatment (Day 0) related to Bray Curtis change from day 0 to day 5 for individual bees. D) Abundance of tet(B) prior to tetracycline treatment (Day 0) related to Jaccard change from day 0 to day 5 for individual bees. E) Abundance of tet(M) prior to tetracycline treatment (Day 0) related to Bray Curtis change from day 0 to day 5 for individual bees. D) Abundance of tet(M) prior to tetracycline treatment (Day 0) related to Jaccard change from day 0 to day 5 for individual bees.

Through our assessment of the abundances of the tetracycline resistance genes tet(B) and tet(M), we found that neither tet(B) nor tet(M) abundance changed significantly from day 0 to day 5 in the control and antibiotic treated bees (Fig 5A and 5B; control tetB, ANOVA, F(1, 12) = 0.01, *p* = 0.918; Eta2 = 9.18e-04, 95% CI [0.00, 1.00]; antibiotic tetB, ANOVA, F(1, 10) = 0.02, *p* = 0.888; Eta2 = 2.10e-03, 95% CI [0.00, 1.00]; control tetM, ANOVA, F(1, 12) = 0.16, *p* = 0.692; Eta2 = 0.01, 95% CI [0.00, 1.00]; antibiotic tetM, ANOVA, F(1, 10) = 3.78, *p* = 0.080; Eta2 = 0.27, 95% CI [0.00, 1.00]). Tet(B) abundance prior to tetracycline exposure did not predict community change from experimental day 0 to day 5 assessed by Bray-Curtis or Jaccard dissimilarity (Fig 5C and 5D; control tetB vs. Bray Curtis distance, linear model, beta = -1.33e-05, 95% CI [-4.69e-05, 2.03e-05], t(5) = -1.02, *p* = 0.357; Std. beta = -0.41, 95% CI [-1.46, 0.63]; antibiotic tetB vs. Bray Curtis distance, linear model, beta = -3.38e-05, 95% CI [-8.00e-05, 1.25e-05], t(4) = -2.03, *p* = 0.113; Std. beta = -0.71, 95% CI [-1.69, 0.26]; control tetB vs. Jaccard distance, linear model, beta = 6.10e-07, 95% CI [-5.34e-05, 5.46e-05], t(5) = 0.03, *p* = 0.978; Std. beta = 0.01, 95% CI [-1.14, 1.16]; antibiotic tetB vs. Jaccard distance, linear model, beta = -1.22e-05, 95% CI [-4.81e-05, 2.37e-05], t (4) = -0.94, *p* = 0.400; Std. beta = -0.43, 95% CI [-1.68, 0.83]). Tet(M) abundance prior to tetracycline exposure did not predict community change based on Bray-Curtis distance, but did predict community change in the antibiotic treated bees based on Jaccard distance (Fig 5E and 5F; control tetM vs. Bray Curtis distance, linear model, beta = 2.41e-04, 95% CI [-2.80e-04, 7.62e-04], t(5) = 1.19, *p* = 0.288; Std. beta = 0.47, 95% CI [-0.55, 1.48]; antibiotic tetM vs. Bray Curtis distance, linear model, beta = -1.59e-03, 95% CI [-4.54e-03, 1.35e-03], t(4) = -1.50, *p* = 0.208; Std. beta = -0.60, 95% CI [-1.71, 0.51]; control tetM vs. Jaccard distance, linear model, beta = 1.78e-04, 95% CI [-6.61e-04, 1.02e-03], t(5) = 0.55, *p* = 0.609; Std. beta = 0.24, 95% CI [-0.88, 1.35]; antibiotic tetM vs. Jaccard distance, linear model, beta = -1.45e-03, 95% CI [-2.38e-03, -5.22e-04], t(4) = -4.34, *p* = 0.012; Std. beta = -0.91, 95% CI [-1.49, -0.33]).

## Discussion

In this study, we present a novel fecal sampling technique for honey bees and demonstrate its effectiveness by utilizing it to examine resistance of the honey bee gut microbiome when challenged by low dose tetracycline disturbance. Fecal sampling is used in other systems (e.g., humans, mice, birds, and wild mammals [60–63]), and is generally considered to mirror the bacterial community of the intestinal tract. We demonstrated that there was little variation in the bacterial gut communities of fecal samples compared to dissected whole gut samples from individual honey bees, when assessed with 16S rRNA amplicon sequencing. Comparisons of beta diversity revealed minimal differences between the fecal communities and whole gut communities collected from the same individual. In addition, we did not find any evidence that individual taxa were differentially represented in the fecal samples. Therefore, the fecal samples accurately represented the bacterial communities found in the intestinal tract of honey bees at the time of collection. Since the honey bee gut microbiome has been proposed as a model system for studies on host-associated bacterial communities [27], allowing the assessment of individual variation over time should be a valuable contribution to future studies.

The majority of insect microbiome studies utilize destructive sampling techniques to assess the structure and function of host associated microbial communities [11,64,65]. Host genetics play a major role in microbiome structure and function [66]. Indeed, even in honey bees, which are all half siblings within a hive, genetic variation shapes the structure of the gut microbiome [67]. Destructive sampling of the microbiome can make it more difficult to account for the effects of intraspecific genetic variation on the microbiome across experimental treatments comprised of different individuals. Additionally, microbiomes are not stagnant, but fluctuate throughout an individual’s life [68,69]. The development and employment of non-destructive microbiome sampling techniques, like the one presented here, will enhance our understanding of how individual microbiomes change over time and respond to variations in the host’s behavior or environment.

We used this fecal sampling method to assess the gut microbiome of individuals pre- and post-exposure to a low dose of tetracycline. In general, we did not see consistent significant change due to the antibiotic treatment, suggesting that, at this low antibiotic dose, these bacterial communities may be resistant to tetracycline disturbance. Previous studies found that tetracycline produces changes in gut community structure and bacterial abundance when bees were fed tetracycline dissolved in sucrose in the lab or when tetracycline was applied to the hive [30,31,70]. Specifically, total bacterial abundance and abundance of core honey bee gut microbiome members decreased in tetracycline treated bees [30,70]. Core members that decreased in abundance when exposed to tetracycline over five days included *Snodgrassella*, *Lactobacillus*, and *Bifidobacterium* [30,31,70]. Raymann et al. [30] and Anderson et al. [70] also found differences in gut communities of control and tetracycline treated bees according to alpha diversity metrics. However, Jia et al. [32] and Baffoni et al. [31] found minimal differences in gut community structure according to alpha diversity metrics following tetracycline treatment, indicating honey bee gut microbiomes may vary in their resistance to tetracycline.

The results we present affirm that the honey bee gut microbiome may be resistant to tetracycline disturbance under certain conditions, and taxa may vary in their susceptibility to tetracycline. Although the gut microbiomes of adult workers are dominated by the same 7–10 bacterial phylotypes, the relative abundance of these phylotypes varies among individuals [71]. This variation may result in qualitative differences among communities from individual bees. Although we did not observe significant mean changes to the gut bacterial communities of tetracycline-treated bees, some of the gut communities appeared to change more in response to exposure. For example, changes in gut community richness, phylogenetic diversity, and Inverse Simpson from day 0 to day 5 varied across individuals, with some communities increasing in diversity, some decreasing, and others remaining essentially the same. We also saw considerable variation in community structure changes among individual bees.

Additionally, the fecal communities from bees in our tetracycline exposure treatment experienced shifts in relative abundance of certain gut taxa after tetracycline treatment. Specifically, *Lactobacillus kunkeei* (ASV11) and the genera *Bombella* had greater relative abundance on day 5 compared to day 0 in the tetracycline treated bees. This finding could either be attributed to these taxa increasing in abundance from day 0 to day 5, or to other taxa within the community becoming less abundant, or to some combination of those changes. Regardless, our results suggest that *Lactobacillus kunkeei* and *Bombella* could be less susceptible to tetracycline; prior literature on these taxa suggests mixed support for this susceptibility. One study examining antimicrobial resistance in *Lactobacillus* strains found a large proportion (>70%) of tetracycline resistance strains in the phylogroup to which *Lactobacillus kunkeei* belongs [72]. But a study on *Bombella* strains suggested most are susceptible to tetracycline [73], although that study was completed in the European Union, where tetracycline is prohibited in beekeeping. The bacterial taxa that did not experience dramatic shifts in relative abundance may have also harbored antibiotic resistance genes. Antibiotic resistance genes have been found in the genomes of many of the core members of the honey bee gut microbiome [34]. Although the hives used in our study were never treated with antibiotics, it is possible the core bacteria contained genes that allowed them to resist tetracycline exposure.

We analyzed the abundance of two tetracycline resistance genes found in honey bee gut members, tet(B) and tet(M), to determine whether the initial abundances of the resistance genes predicted tetracycline resistance. We found that tet(M) abundance prior to tetracycline treatment was negatively correlated with Jaccard distance in the tetracycline-treated bees, indicating that tet(M) may provide some resistance to honey bee bacterial taxa that prevents change in community membership when exposed to tetracycline. In addition, we determined whether resistance gene abundance was influenced by tetracycline treatment. We predicted the bacterial communities would adapt to tetracycline treatment through the proliferation of tetracycline resistance genes. Indeed, Baffoni et al. [31] observed greater abundances of the tetracycline resistance genes tet(W) and tet(Y) within honey bee gut microbiomes following tetracycline exposure. For both tet(B) and tet(M) we did not see considerable changes in abundance following tetracycline treatment. The lack of change we observed could be attributed to the low tetracycline dose used in this study, or our relatively small sample size. Alternatively, the differentially abundant taxa may have possessed other tetracycline resistance genes, as there are more than 60 genes that confer resistance to tetracycline [74].

Overall, we did not find much evidence that low doses of tetracycline significantly alter the honey bee gut microbiome, however, our results suggest gut communities from individual honey bees, as well as certain honey bee symbionts, may vary in resistance to tetracycline. The presence of tetracycline resistance genes within microbiome members may contribute to gut microbiome stability, and certain members like *Gilliamella* may disproportionately harbor tetracycline resistance genes. In future studies, metagenomic or proteomic analyses may help to further our understanding of how antibiotic resistance genes and other genetic components contribute to microbiome stability. In addition, we suggest that fecal sampling in honey bees could be used to track gut microbiome community structure in individuals over time. This opens up many opportunities to study changes in microbiome structure over time, compare host-microbiome dynamics among individuals, and analyze how host associated microbial communities respond to disturbance.

## Supporting information

S1 Table ALDEx2 column descriptions. Column descriptions for ALDEx2 outputs.
(CSV)

S2 Table ALDEx2 results for differentially abundant amplicon sequence variants (ASVs). ALDEx2 output comparing differentially abundant ASVs in the antibiotic treated bees before and five days after treatment with 4.5 μg tetracycline.
(CSV)

S3 Table ALDEx2 results for differentially abundant genera. ALDEx2 output comparing differentially abundant genera in the antibiotic treated bees before and five days after treatment with 4.5 μg tetracycline.
(CSV)
